# Acute effects of empagliflozin on open-loop baroreflex function and urinary glucose excretion in rats with chronic myocardial infarction

**DOI:** 10.1186/s12576-023-00877-1

**Published:** 2023-09-13

**Authors:** Toru Kawada, Meihua Li, Akitsugu Nishiura, Yuki Yoshida, Shohei Yokota, Hiroki Matsushita, Masafumi Fukumitsu, Kazunori Uemura, Joe Alexander, Keita Saku

**Affiliations:** 1 Department of Cardiovascular Dynamics, National Cerebral and Cardiovascular Center, Osaka, 564-8565 Japan; 2 Medical and Health Informatics, NTT Research, Inc, Sunnyvale, CA 94085 USA

**Keywords:** Sodium–glucose cotransporter 2, Sympathetic nerve activity, Arterial pressure, Urine flow, Equilibrium diagram

## Abstract

Sodium–glucose cotransporter 2 (SGLT2) inhibitors have exerted cardioprotective effects in clinical trials, but underlying mechanisms are not fully understood. As mitigating sympathetic overactivity is of major clinical concern in the mechanisms of heart failure treatments, we examined the effects of modulation of glucose handling on baroreflex-mediated sympathetic nerve activity and arterial pressure regulations in rats with chronic myocardial infarction (*n* = 9). Repeated 11-min step input sequences were used for an open-loop analysis of the carotid sinus baroreflex. An SGLT2 inhibitor, empagliflozin, was intravenously administered (10 mg/kg) after the second sequence. Neither the baroreflex neural nor peripheral arc significantly changed during the last observation period (seventh and eighth sequences) compared with the baseline period although urinary glucose excretion increased from near 0 (0.0089 ± 0.0011 mg min^−1^ kg^−1^) to 1.91 ± 0.25 mg min^−1^ kg^−1^. Hence, empagliflozin does not acutely modulate the baroreflex regulations of sympathetic nerve activity and arterial pressure in this rat model of chronic myocardial infarction.

## Background

Sodium–glucose cotransporter 2 (SGLT2) inhibitors were developed to treat diabetes mellitus (DM), but they are now recognized as a new class of drugs to treat heart failure [1, 2]. The cardioprotective effect of SGLT2 inhibitors has been demonstrated in several clinical trials (DAPA-HF [3], EMPEROR-Reduced [4], EMPEROR-Preserved [5]), but its exact underlying mechanisms of action are not fully understood. The mechanisms may be multifactorial, including glucotoxicity attenuation, improved cardiac loading conditions via the body fluid volume reduction, and improvements in cardiac metabolism [6–8]. Although the sympathoinhibitory effect of SGLT2 inhibitors may also help facilitate cardioprotection [9–13], whether SGLT2 inhibitors acutely modify systemic sympathetic nerve activity (SNA) remains to be clarified. SGLT2 is primarily expressed in renal proximal tubules [14], thus the potential sympathoinhibitory effect may be related to the suppression of sympathoexcitatory reflexes from the kidneys. Renal sensory nerve activation can yield sympathoexcitatory reflexes in diseased kidneys while eliciting inhibitory renorenal reflexes in normal kidneys [15, 16].

Glucose reabsorption via SGLT2 depends on the sodium gradient across the plasma membrane, which is maintained by Na^+^/K^+^ ATPase activity. Hence, glucose reabsorption is energy consuming. The ATP level in the renal proximal tubules decreases shortly after renal ischemia [17], indicating a high metabolic rate and limited ATP storage in proximal tubular cells. Increased organ work or oxygen deficiency may cause renal stress, thereby activating sympathoexcitatory reflexes. The glucose reabsorption by the proximal tubules increases as the filtered load is increased by either an elevation in plasma glucose concentration or an increase in glomerular filtration rate until it reaches a threshold [18]. Increases in the filtered load above this maximal reabsorptive capacity results in glucosuria. Hence, DM represents a pathological situation of increased organ work with the proximal tubular cells working at their maximum reabsorptive capacity. We have examined the acute effect of an SGLT2 inhibitor, empagliflozin, on the baroreflex open-loop static characteristics in Goto–Kakizaki diabetic rats [19]. The study revealed that intravenous empagliflozin administration did not acutely attenuate SNA despite a significantly increased urinary glucose excretion. Therefore, the possible sympathoinhibitory effect of empagliflozin may be an indirect effect associated with chronic improvements in renal energy status and general disease conditions.

The present study used a rat model of chronic myocardial infarction (MI) to test the hypothesis that empagliflozin acutely mitigates systemic sympathoexcitation. We employed an open-loop analysis of the carotid sinus baroreflex [20] because this method allows us to test the drug effect on SNA over a wide range of carotid sinus pressure (CSP). For instance, the sympathoinhibitory effect of a central antihypertensive, clonidine, is more evident in the low CSP (high SNA) range than in the high CSP (low SNA) range [21]. Generally speaking, heart failure causes renal hypoperfusion and induces potential tubular damages [22, 23]. If renal dysfunction in post-MI rats causes centrally-mediated systemic sympathoexcitation via renal afferent nerves, the inhibition of SGLT2 might reduce energy consumption at the kidneys and attenuate the sympathoexcitatory reflexes, resulting in systemic sympathetic suppression.

## Methods

### Ethical approval

Male Wistar–Kyoto (WKY) rats were purchased from Japan SLC. The rats were cared for strictly following the Guiding Principles for the Care and Use of Animals in the Field of Physiological Sciences, which have been approved by the Physiological Society of Japan. The Animal Subjects Committee at the National Cerebral and Cardiovascular Center reviewed and approved the experimental protocols (No. 22033). As a reference, the data of body weight and heart weight obtained from age-matched WKY rats used in another study (No. 23013) were reported. We also included the previously reported data of plasma creatinine concentration obtained from WKY rats with unilateral renal denervation [24] and Goto-Kakizaki diabetic rats [19].

### Preparation

The proximal left coronary artery (before branching into the anterior descending and circumflex arteries) was permanently ligated with a 5–0 Prolene suture (Ethicon, Johnson & Johnson, NJ, USA) at 8 weeks of age following a previously described procedure [25]. Of 30 rats that underwent coronary ligation, 12 survived for > 8 weeks and were subjected to an acute experiment described below, and the data from 9 rats were eventually analyzed because of the incomplete dataset.

The rats were anesthetized with an intraperitoneal injection (2 mL/kg) of a urethane (250 mg/mL) and α-chloralose (40 mg/mL) mixture. The anesthetic mixture was diluted 18-fold with physiological saline and continuously infused via the right femoral vein (2 mL·kg^−1^ h^−1^). Ringer’s lactate solution was continuously infused (4 mL kg^−1^ h^−1^) to maintain fluid balance. A respirator was used to ventilate the animals with oxygen-enriched air. Arterial pressure (AP) was measured from the right femoral artery. A cardiotachometer (AT-601G, Nihon Kohden, Japan) was used to determine the heart rate (HR) from the AP waveform. Central venous pressure (CVP) was measured via a catheter inserted into the left femoral vein and advanced to the inferior vena cava. Body temperature was maintained between 37 °C and 38 °C using a heating pad and a lamp.

A left flank incision exposed the postganglionic branch of the splanchnic sympathetic nerve. A pair of stainless-steel wire electrodes (AS633, Cooner Wire, CA, USA) was attached to the nerve and fixed with silicone glue (Kwik-Sil, World Precision Instruments, FL, USA). The electrical signal was band-pass filtered between 150 and 1000 Hz, full-wave rectified, and low-pass filtered at a cut-off frequency of 30 Hz. A ganglionic blocker, hexamethonium bromide (60 mg/kg), was administered at the end of the experiment to assess the noise level of SNA recording. The splanchnic SNA was chosen as a proxy of systemic SNA because the splanchnic vascular bed plays a substantial role in the systemic circulatory control [26].

The bilateral carotid sinus baroreceptor regions were isolated from the systemic circulation [27, 28], and a servo-controlled piston pump system (ET-126, Labworks, CA, USA) externally regulated CSP. Reflexes other than the carotid sinus baroreflex were minimized by sectioning the aortic depressor and vagal nerves in the neck region.

Urine was collected via polyethylene tubes (KN-392-SP 8, Natsume, Japan) that were inserted into bilateral ureters through a horizontal abdominal incision. The urine was accumulated on a balance dish, and urine volume (UV) was gravitationally assessed. The readout (resolution: 0.1 mg ≈ 0.1 μL) of an electronic balance (HR-150AZ, A&D Company, Japan) was recorded in synchrony with the hemodynamic data [29].

The left renal artery was isolated by gently dissecting the surrounding tissues. An ultrasonic flow probe (MA1PRB, Transonic Systems Inc., NY, USA) was attached to the renal artery to measure renal blood flow (RBF).

### Protocol

CSP was decreased to 60 mmHg for five min and then increased stepwise up to 180 mmHg in increments of 20 mmHg every minute. The stepwise CSP input sequence was repeated and denoted as S1 through S8. Empagliflozin (MedChemExpress, NJ, USA) was dissolved in dimethyl sulfoxide (DMSO) at 10 mg/100 μL and diluted with polyethylene glycol and physiological saline to a final concentration of 10 mg/mL (10% v/v DMSO, 45% v/v polyethylene glycol 200, and 45% v/v physiological saline). The empagliflozin solution was intravenously administered at 10 mg/kg (1 mL/kg, bolus) at 1 min after S2 completion [19].

### Data analysis

The CSP, SNA, AP, HR, RBF, CVP, and UV data were stored on a laboratory computer system at 1000 Hz via a 16-bit analog-to-digital converter. Data were averaged during the last 10 s of each step for each of the S1–S8 sequences to assess the open-loop static characteristics of the carotid sinus baroreflex. Renal vascular resistance (RVR) was derived from RVR = (AP − CVP)/RBF. Urine flow (UF) (in μL/min) was derived from a 1-min increment of UV. Then, normalized UF (nUF) (in μL min^−1^ kg^−1^) was calculated from UF divided by the body weight of the rat. Urine samples were combined for two consecutive sequences for urinary glucose, sodium, and creatinine concentration measurements. The S1 and S2 sequences are referred to as the baseline (BL) period. The S3 and S4, S5 and S6, and S7 and S8 are referred to as T1, T2, and T3 periods, respectively. Further, the hemodynamic data are reported as averaged values for BL, T1, T2, and T3 periods.

The SNA was normalized in each animal because the absolute amplitude of SNA significantly varied across animals depending on recording conditions. The value obtained after the ganglionic blockade was defined as 0%, and the value corresponding to the CSP of 60 mmHg during BL was defined as 100%. Regardless of the normalization, the acute effect of empagliflozin on SNA, if any, can be detected as the relative change from the BL value.

A four-parameter logistic function (Eq. 1) quantified the static characteristics of the baroreflex total arc (AP as a function of CSP), HR control (HR as a function of CSP), and the neural arc (SNA as a function of CSP) [20, 30]:

1 $$y = \frac{{P_{1} }}{{1 + exp\left[ {P_{2} \left( {CSP - P_{3} } \right)} \right]}} + P_{4}$$

where *P*_1_ is the response range; *P*_2_ is the slope coefficient; *P*_3_ is the midpoint pressure on the CSP axis; *P*_4_ is the lower asymptote of the sigmoid curve.

Linear regression quantified the static characteristics of the baroreflex peripheral arc (AP as a function of SNA) (Eq. 2) [20]:

2 $$y = b_{0} + b_{1} x$$

where *b*_0_ and *b*_1_ denote the intercept and slope, respectively. The SNA–RVR relationship was likewise analyzed using linear regression.

The baroreflex equilibrium diagram was obtained by plotting the neural and peripheral arcs on a pressure versus SNA plane [31, 32]. The operating-point SNA and AP values were determined at the intersection point of the fitted neural and peripheral arcs on the equilibrium diagram.

The AP–nUF relationship during a stepwise CSP input approximated a straight line in our previous studies [24, 29, 33]. The intercept (nUF-intercept) and slope (nUF-slope) were estimated using linear regression. In addition, the following logarithmic function was adopted to quantify the AP–nUF relationship because the relationship was slightly curvilinear in the lower AP range in the present dataset:

3 $$y = G\,{\text{log}}_{10} \left( {x - x_{0} } \right)$$

where log_10_(·) is the common logarithm; *G* and *x*_0_ denote the gain and x-intercept, respectively.

### Blood and urine samples

An arterial blood sample was obtained once at the end of the experiment. The plasma sample was frozen at −80 °C after centrifugation. Urine samples obtained during BL, T1, T2, and T3 periods were also frozen at −80 °C. Later, the glucose, sodium, and creatinine concentrations were measured by outsourcing (SRL Inc., Japan).

The urinary glucose and sodium excretions were calculated from the product of the average nUF and the urinary glucose and sodium concentrations, respectively. Creatinine clearance (C_cr_) was calculated as the value per kidney by halving the average nUF for compatibility with our previous reports [19, 24, 33, 34]. The plasma creatinine concentration was assumed to be unchanged significantly throughout the experiment.

### Statistical analysis

The final analysis excluded data from three rats due to trouble in the UV measurement and failure to obtain stable SNA and CVP recordings, respectively. Eventually, data from nine post-MI rats were analyzed. All data are expressed as mean ± standard error (SE) values. Time-dependent changes in the baroreflex static characteristic parameters, urinary excretion-related data, and AP–nUF relationship parameters were analyzed using one-way repeated-measures analysis of variance (ANOVA) with the Greenhouse–Geisser correction, followed by Dunnett’s test (Prism 8, GraphPad Software, CA, USA). A nonparametric Mann–Whiney test [35] compared the plasma creatinine concentration with the values available from our previous reports, as a reference. *P*-values of < 0.05 in all the statistical analyses were considered significant differences.

## Results

Table 1 summarizes the body weight, heart weight (biventricular weight), and normalized heart weight. The post-MI rats demonstrated a range of cardiac remodeling. The absolute heart weight and normalized heart weight were significantly heavier in post-MI rats than those in age-matched normal WKY rats subjected to experiments different from the present study (*n* = 5, age: 17.5 ± 0.7 weeks, *P* = 0.606; heart weight: 0.903 ± 0.018 g, *P* = 0.004; normalized heart weight: 2.251 ± 0.043 g/kg, *P* < 0.001 by Mann–Whitney test). Further, Table 1 summarizes the concentrations of plasma glucose, sodium, and creatinine measured from the arterial blood sampled at the end of the experiment. The plasma creatinine concentration was significantly higher in post-MI rats than that in WKY rats with unilateral renal denervation [24] (*n* = 9, 0.39 ± 0.02 pg/mL, *P* < 0.001 by Mann–Whitney test) or that in Goto–Kakizaki rats [19] (*n* = 7, 0.33 ± 0.05 pg/mL, *P* = 0.002 by Mann–Whitney test).

**Table 1:** Age, body weight, heart weight, and plasma levels of glucose, sodium, and creatinine

	Mean ± SE	Minimum	Maximum
Age (weeks)	18.1 ± 0.4	16.1	20.6
Body weight (g)	423 ± 9	377	462
Heart weight (g)	1.144 ± 0.064	0.924	1.596
Normalized heart weight (g/kg)	2.69 ± 0.10	2.45	3.45
Plasma glucose (mg/dL)	214 ± 13	166	281
Plasma sodium (mEq/L)	139 ± 1	134	142
Plasma creatinine (mg/dL)	0.61 ± 0.03	0.51	0.81

Data are derived from nine post-myocardial infarction rats. Plasma glucose, sodium, and creatinine concentrations were measured from the arterial blood sample obtained at the end of the experiment. *SE* standard error

**Fig. 1:**
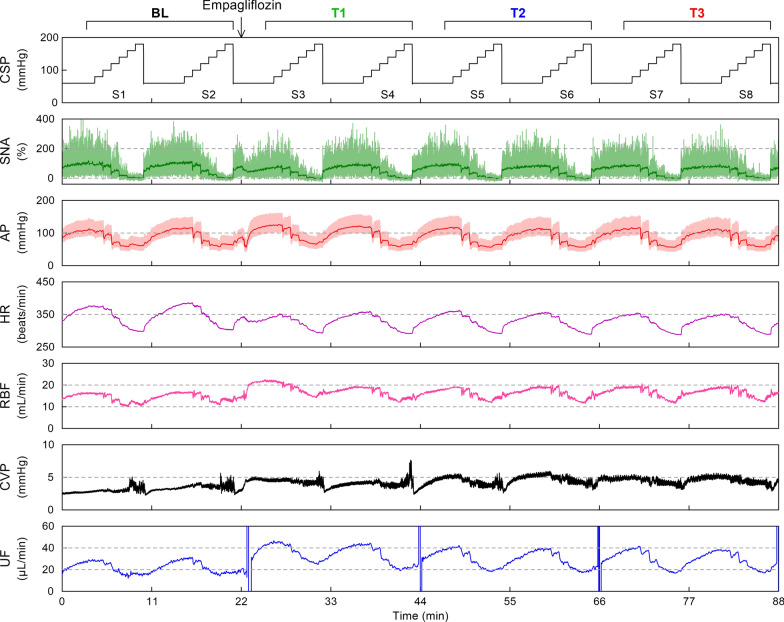
An example time series obtained from a rat with chronic myocardial infarction. Carotid sinus pressure (CSP), sympathetic nerve activity (SNA), arterial pressure (AP), heart rate (HR), renal blood flow (RBF), central venous pressure (CVP), and urine flow (UF) during stepwise input sequences (S1–S8) are shown. The UF was derived from the time derivative of the urine volume signal. The CSP plot shows the 10-Hz resampled signal. Shown are 10-Hz resampled (pale green) and 2-s moving averaged (dark green) signals in the SNA plot, as well as 100-Hz resampled (pale red) and 2-s moving averaged (red) signals in the AP plot. The HR, RBF, CVP, and UF plots show 2-s moving averaged signals. Data were divided into baseline (BL), and the first (T1), second (T2), and third (T3) periods after empagliflozin administration. The acute vertical changes in the UF plot are artifacts due to manual urine removals from the balance dish. The empagliflozin solution was administered 1 min after S2 completion (downward allow)

Figure 1 illustrates an example of time series obtained from one post-MI rat. The stepwise increase in CSP suppressed SNA, followed by AP and HR reductions. RBF decreased as the AP decreased in this animal. CVP did not decrease with decreasing AP but demonstrated fluctuations during low AP levels. The low AP might have caused respiratory center hypoperfusion and induced spontaneous respiratory activity, which affected CVP through intrathoracic pressure changes. Empagliflozin administration caused slight SNA, AP, and HR decreases, but the effects were transient. The SNA responses during T1, T2, and T3 were not significantly different from that during BL. The maximum AP increased during T1, but the increase subsided during T3. HR decreased, whereas RBF and CVP slightly increased after empagliflozin administration in this animal. Acute vertical changes in the UF plot were artifacts due to manual urine removals. The UF demonstrated near parallel change with AP, and the mean levels and amplitude significantly increased after empagliflozin.

**Fig. 2:**
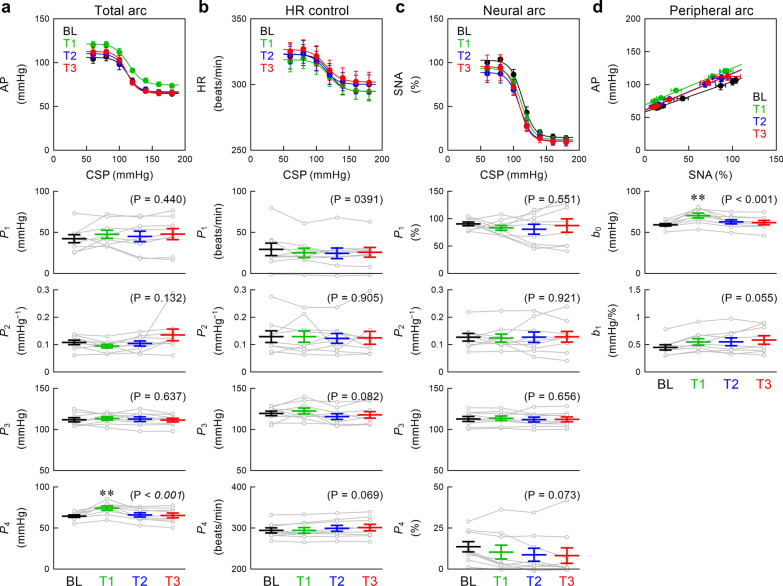
Static characteristics of the baroreflex total arc (**a**), HR control (**b**), and neural arc (**c**) were analyzed using a four-parameter logistic function. Static characteristics of the baroreflex peripheral arc (**d**) were analyzed using linear regression. Data were obtained during the baseline (BL) period and after empagliflozin administration (T1, T2, and T3 periods). AP: arterial pressure; CSP: carotid sinus pressure; HR: heart rate; SNA: sympathetic nerve activity; *P*_1_, *P*_2_, *P*_3_, and *P*_4_ represent the response range, slope coefficient, midpoint pressure, and lower asymptote of the sigmoid curve, respectively (Eq. 1); *b*_0_ and *b*_1_ represent the intercept and slope of linear regression, respectively (Eq. 2). Data points show mean ± standard error values (n = 9 post-myocardial infarction rats) with parameters of respective rats illustrated in gray lines. The P values in the parentheses represent the results of one-way repeated-measures analysis of variance. **P < 0.01 relative to BL by the post hoc Dunnett’s test

Figure 2 summarizes the group-averaged static characteristics of the carotid sinus baroreflex. Empagliflozin increased the lower asymptote of the total arc without affecting other parameters during T1 (Fig. 2a). Empagliflozin did not significantly affect any of the fitted parameters in the HR control (Fig. 2b) and the neural arc (Fig. 2c). Empagliflozin significantly increased the intercept of the peripheral arc during T1 (Fig. 2d). The slope of the peripheral arc tended to be different among the four periods, with the mean value increasing with time.

**Fig. 3:**
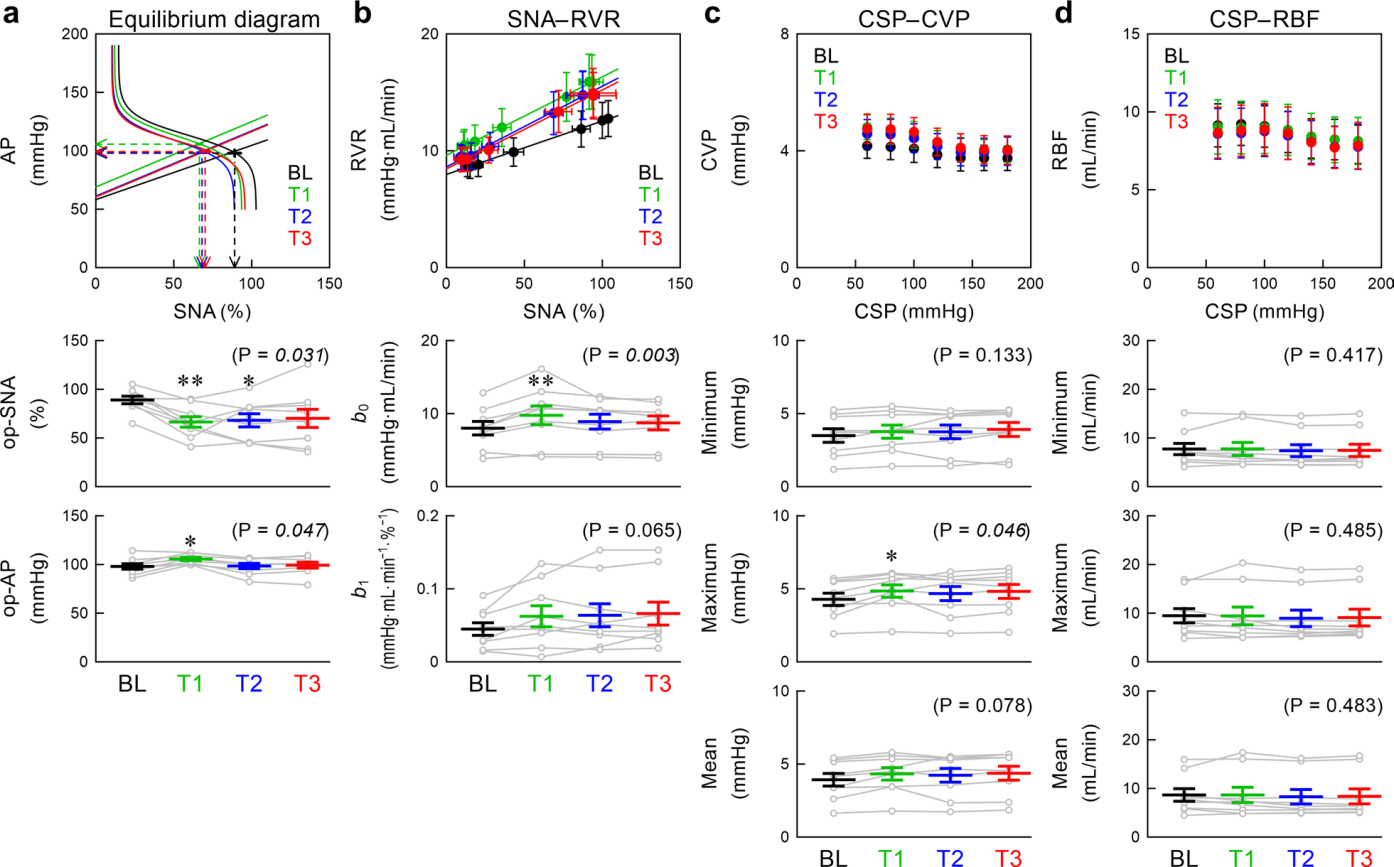
The baroreflex equilibrium diagram was obtained by plotting the fitted neural and peripheral arcs on the pressure–SNA plane (**a**). The SNA–RVR relationship was quantified using linear regression (**b**). For the CSP–CVP (**c**) and CSP–RBF (**d**) relationships, the minimum, maximum, and mean values were examined because the relationships were not consistent across the rats. Data were obtained during the baseline (BL) period and after empagliflozin administration (T1, T2, and T3 periods). AP: arterial pressure; CSP: carotid sinus pressure; CVP: central venous pressure; RBF: renal blood flow; RVR: renal vascular resistance; SNA: sympathetic nerve activity; op-SNA and op-AP represent the operating-point SNA and AP, respectively; *b*_0_ and *b*_1_ represent the intercept and slope of linear regression, respectively. Data points show mean ± standard error values (*n* = 9 post-myocardial infarction rats) with parameters of respective rats illustrated in gray lines. The P values in the parentheses represent the results of one-way repeated-measures analysis of variance. *P < 0.05 and **P < 0.01 relative to BL by the post hoc Dunnett’s test

In the baroreflex equilibrium diagram (Fig. 3a), the operating-point SNA (the downward arrowhead) decreased after empagliflozin, and the difference from BL was statistically significant during T1 and T2. The operating-point AP (the leftward arrowhead) increased during T1 relative to BL. The SNA–RVR relationship approximated a straight line (Fig. 3b). Empagliflozin increased the intercept during T1, but the effect waned during T2 and T3. The slope of the SNA–RVR relationship tended to be higher after empagliflozin compared with that in BL. The CVP response was not consistent across the animals although the mean data points of the CSP–CVP relationship distributed in an inverse sigmoidal fashion (Fig. 3c). Empagliflozin significantly increased the maximum CVP during T1 relative to BL. The mean data points of the CSP–RBF relationship also distributed in an inverse sigmoid fashion (Fig. 3d), but the RBF response was not consistent across the animals. Empagliflozin did not significantly affect the minimum, maximum, or mean RBF.

**Fig. 4:**
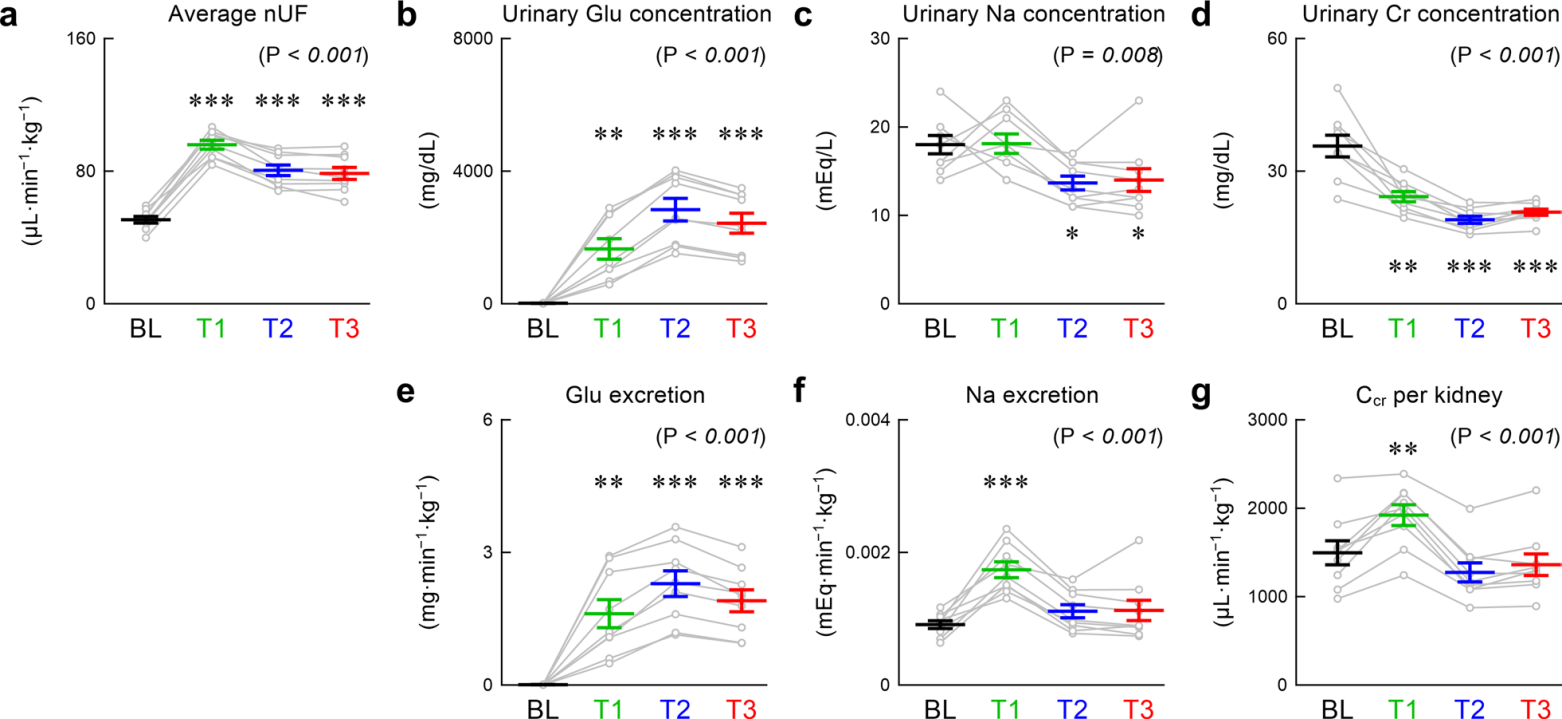
Time course of average normalized urine flow (nUF) (**a**), urinary glucose (Glu) concentration (**b**), urinary sodium concentration (**c**), urinary creatinine (Cr) concentration (**d**), urinary Glu excretion (**e**), urinary sodium excretion (**f**), and creatinine clearance (C_cr_) per kidney (**g**). Data were obtained during the baseline (BL) period and after empagliflozin administration (T1, T2, and T3 periods). Data are mean ± standard error and individual rats (n = 9 post-myocardial infarction rats). The P values in the parentheses indicate the results of one-way repeated-measures analysis of variance. *P < 0.05, **P < 0.01, and ***P < 0.001, relative to BL by post hoc Dunnett’s test

Figure 4 presents urinary excretion-related data. The average nUF (Fig. 4a) increased after empagliflozin. The nUF peaked during T1 and remained increased until the end of the observation period. The urinary glucose concentration was 17.4 ± 2.0 mg/dL during BL and markedly increased after empagliflozin (Fig. 4b). The urinary sodium concentration was not significantly different between BL and T1 but decreased during T2 and T3 compared with BL (Fig. 4c). The urinary creatinine concentration significantly decreased after empagliflozin (Fig. 4d). The glucose excretion was near 0 (0.0089 ± 0.0011 mg·min^−1^ kg^−1^) during BL and markedly increased after empagliflozin (Fig. 4e). The sodium excretion significantly increased during T1 but returned toward the BL value during T2 and T3 (Fig. 4f). The C_cr_ per kidney transiently increased during T1. The mean value of C_cr_ decreased during T2 and T3 relative to BL although the difference was not statistically significant in the post hoc analysis (Fig. 4g).

**Fig. 5:**
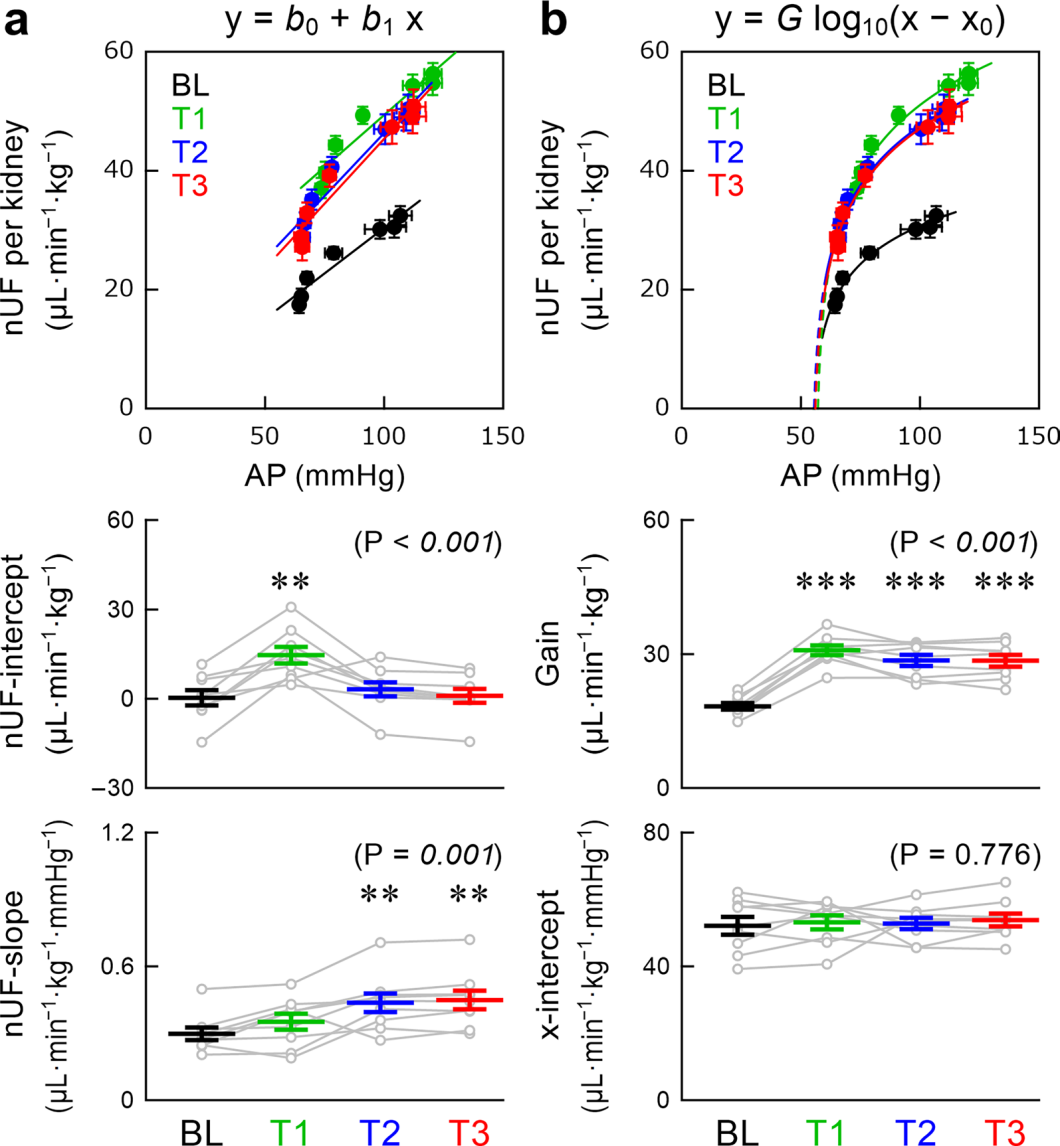
The relationship between arterial pressure and normalized urine flow (nUF) per kidney was analyzed with linear regression (**a**) and logarithmic function (**b**). The linear regression indicated *b*_0_ and *b*_1_ as the nUF-intercept and nUF-slope, respectively. The analysis using the logarithmic function indicated *G* and x_0_ as the gain and the x-intercept, respectively. Data were obtained during the baseline (BL) period and after empagliflozin administration (T1, T2, and T3 periods). Data are mean ± standard error and individual rats (*n* = 9 post-myocardial infarction rats). The P values in the parentheses indicate the results of one-way repeated-measures analysis of variance. **P < 0.01 and ***P < 0.001, relative to BL by post hoc Dunnett’s test

Figure 5 illustrates the relationship between AP and nUF per kidney during the stepwise changes in CSP. For the linear regression analysis (Fig. 5a), empagliflozin significantly increased the nUF-intercept during T1, but the increasing effect was not seen during T2 and T3. Instead, empagliflozin significantly increased the nUF-slope during T2 and T3 relative to BL. The R^2^ values between linearly estimated and measured nUF values were 0.883 ± 0.028, 0.887 ± 0.030, 0.910 ± 0.020, and 0.918 ± 0.020 for BL, T1, T2, and T3 periods, respectively. For the logarithmic function analysis (Fig. 5b), the gain significantly increased after empagliflozin throughout the observation period. The intercept on the AP axis was approximately 55 mmHg for all of the BL, T1, T2, and T3 periods. The R^2^ values between logarithmically estimated and measured nUF values were 0.912 ± 0.024 (P = 0.074), 0.931 ± 0.025 (P = 0.004), 0.940 ± 0.022 (*P* = 0.027), and 0.949 ± 0.020 (P = 0.027), respectively, where the P values were calculated between the linear and logarithmic estimations using Wilcoxon signed-rank test.

## Discussion

The acute effects of an intravenous administration of empagliflozin on baroreflex function and urinary excretion were examined in post-MI rats. The baroreflex neural arc, which describes the baroreflex-mediated SNA response over a wide CSP range, remained unchanged while the effect of empagliflozin on urinary glucose excretion was confirmed during the entire observation period. There was a transient increase in the intercept of the peripheral arc after the empagliflozin administration. During the last observation period, AP did not change relative to BL despite the increased UF.

### Effects of empagliflozin on open-loop baroreflex function

Renal energy status can affect systemic SNA via renal afferent signaling [9]. For instance, adenosine-sensitive nerve endings in the renal pelvis increase systemic SNA to produce hypertension in one-kidney, one-clip rats [36]. Heart failure may predispose the kidneys to oxygen deficiency because of decreased cardiac output as well as RBF [22, 23]. The post-MI rats in the present study demonstrated a moderately increased plasma creatinine concentration, indicating renal injury development. The expected SNA inhibition was not detected in the baroreflex neural arc (Fig. 2c) although the SGLT2 inhibition reduces ATP consumption associated with glucose reabsorption at renal proximal tubules. The result is consistent with our previous study on Goto–Kakizaki diabetic rats demonstrating a lack of acute neural arc modulation after empagliflozin administration [19]. The sympathoinhibitory effect of empagliflozin may not be acute but chronic and indirect through renal protection and general disease condition improvement. A study by Gueguen et al. [12] revealed that 1-week empagliflozin treatment, but not a single acute dose, attenuated the exaggerated sympathetic response to lowering AP in diabetic rabbits.

The lower asymptote of the total arc increased during T1 (Fig. 2a), with an increased AP-intercept of the peripheral arc (Fig. 2d). This increase might have partly resulted from a volume loading effect of the empagliflozin administration because of the increased maximum CVP during T1 (Fig. 3c). Changes in RVR might have also contributed to the increase in the AP-intercept of the peripheral arc. The mean SNA–RVR relationship displaced upward during T2 and T3 relative to BL (Fig. 3b) although the difference in the fitted parameters was not statistically significant. The increased RVR after empagliflozin may be mediated by tubuloglomerular feedback (TGF) [8]. SGLT2 inhibition at proximal tubules increases the amount of sodium reaching the macula densa of the distal tubules. This information causes constriction of afferent arterioles to protect the glomeruli by reducing the intraglomerular pressure. The present study revealed an increased amount of urinary sodium excretion during T1 (Fig. 3f), possibly induced afferent arteriole constriction through TGF. Whether vascular resistance in other body areas increases after empagliflozin administration remains unanswered in the present study. Empagliflozin can relax resistance mesenteric arteries in vitro [37]. However, the peripheral arc should have displaced downward if the vasodilative effect of empagliflozin was dominant, as in the case with an angiotensin II type 1 receptor blocker, telmisartan [34] or a soluble guanylate cyclase stimulator, vericiguat, administration [38]. Another factor contributing to AP maintenance after empagliflozin administration is the differential volume regulation between interstitial and intravascular fluid volume by SGLT2 inhibitors [39]. SGLT2 inhibitors reduce the insulin:glucagon ratio that increases hepatic glucose production [40, 41]. The increased hepatic glucose production moves the water from the interstitial space or cells into the vessels because water moves with glucose [42].

The upward peripheral arc displacement may have increased the cardiac afterload and is disadvantageous for the failing heart. However, hemodynamics were not acutely deteriorated after empagliflozin administration in the present study. Recently, a direct cardiac effect of empagliflozin to improve cardiac energy status is suggested despite the lack of SGLT2 expression in cardiac tissues [43]. Such an off-target effect of empagliflozin might have helped the failing heart better tolerate the increased afterload. A slight peripheral arc improvement, i.e., an increase in the slope and/or the intercept of the SNA–AP relationship, caused a marked operating-point SNA reduction as the sigmoid curve of the neural arc intersects with the peripheral arc near the upper plateau in post-MI rats compared with normal rats [44]. The baroreflex equilibrium diagram predicted a significantly reduced operating-point SNA with a slight increase (T1) and no change (T2) in the operating-point AP, though these changes became statistically insignificant during the last observation period (T3) (Fig. 3a). The present finding indicates that keeping AP slightly above the baroreflex threshold pressure could reduce systemic SNA and might help terminate the vicious circle of heart failure associated with sympathetic overactivity. Mechanical circulatory support might achieve somewhat exclusive goals of elevating AP while unloading the failing heart [45]. Instead of elevating AP, stimulating baroreceptors is explored as an alternative approach to treat heart failure [46].

### Effects of empagliflozin on urinary excretion

AP increases with SNA in the present experimental settings (Fig. 2d), and AP positively correlated with nUF (Fig. 5) although sympathetic activation exerts an antidiuretic effect through renal vasoconstriction, renin release, and promotion of sodium and water reabsorption [47]. Hence, pressure diuresis outweighs the neurally mediated antidiuretic effect [29]. Empagliflozin increased the mean nUF, thereby facilitating extracellular fluid volume normalization and relieving heart failure-related congestion. Additionally, empagliflozin increased the slope or gain of the AP–nUF relationship (Fig. 5), thereby allowing finer control of extracellular fluid volume in response to AP changes. The slope of the AP–nUF relationship became steeper toward the lower AP range, yielding a convex curvilinearity in the post-MI rats, although the AP–nUF relationship was more linear in our previous studies on normal WKY rats [24, 29]. The x-intercept of the logarithmic function may point to the minimum AP necessary to keep urine production (Fig. 5b). This nonlinearity probably helps avoid excess fluid loss after empagliflozin administration.

## Limitations

We only examined the acute effects of empagliflozin under anesthetized conditions while MI was induced chronically. Hence, the results cannot directly be extrapolated to an interpretation of the long-term effect of empagliflozin in a conscious state. As we did not test empagliflozin on sham-operated rats, it remains uncertain whether the observed results were specific to chronic MI rats. Second, the surrounding structures, including the renal nerves, could be damaged to some extent during the flow probe attachment to the left renal artery. Hence, the results of RBF and RVR need to be carefully interpreted. Third, cardiac function was not assessed in the present study. A continuous, stable recording of left ventricular pressure is challenging in the present experimental setting because the large change in the cardiac loading condition can induce ectopic beats possibly through contact of a catheter tip with the ventricular wall. The measurements of cardiac output and left atrial pressure in addition to CVP (≈ right atrial pressure) may allow us to estimate changes in stressed blood volume [48]. Further studies are required to quantify the relationship between changes in UF and stressed blood volume after empagliflozin. Fourth, the vagal nerves were sectioned to establish the open-loop condition of the carotid sinus baroreflex. Although we did not measure cardiac SNA, the HR data may roughly reflect changes in cardiac SNA because of vagotomy. The HR did not decrease significantly after empagliflozin, indicating that empagliflozin did not significantly affect cardiac SNA. Direct measurements of renal afferent and efferent nerve activities are also desirable to strengthen the discussion, though the present results did not support acute modulation of sympathetic regulation by empagliflozin.

## Conclusions

The effect of empagliflozin on open-loop baroreflex function was examined in post-MI rats. The sympathoinhibitory effect of empagliflozin was not detected within the observation period. AP, at a given SNA, was well maintained despite a significant increase in urine excretion. Empagliflozin would take more time to manifest a significant hemodynamic effect in this rat model of chronic MI.

## Data Availability

The datasets used and/or analyzed during the current study are available from the corresponding author on reasonable request.

## Abbreviations

AP: Arterial pressure
BL: Baseline
C_cr_: Creatinine clearance
CSP: Carotid sinus pressure
CVP: Central venous pressure
DM: Diabetes mellitus
DSMO: Dimethyl sulfoxide
HR: Heart rate
MI: Myocardial infarction
nUF: Normalized urine flow
RBF: Renal blood flow
RVR: Renal vascular resistance
SGLT2: Sodium–glucose cotransporter 2
SNA: Sympathetic nerve activity
TGF: Tubuloglomerular feedback
UF: Urine flow
UV: Urine volume
WKY: Wistar–Kyoto

## References

[1] Zinman B, Wanner C, Lachin JM, Fitchett D, Bluhmki E, Hantel S, Mattheus M, Devins T, Johansen OE, Woerle HJ, Broedl UC, Inzucchi SE, Investigators EMPA-REGOUTCOME (2015). Empagliflozin, cardiovascular outcomes, and mortality in type 2 diabetes. N Engl J Med.

[2] Bauersachs J (2021). Heart failure drug treatment: the fantastic four. Eur Heart J.

[3] McMurray JJV, Solomon SD, Inzucchi SE, Køber L, Kosiborod MN, Martinez FA, Ponikowski P, Sabatine MS, Anand IS, Bělohlávek J, Böhm M, Chiang CE, Chopra VK, de Boer RA, Desai AS, Diez M, Drozdz J, Dukát A, Ge J, Howlett JG, Katova T, Kitakaze M, Ljungman CEA, Merkely B, Nicolau JC, O'Meara E, Petrie MC, Vinh PN, Schou M, Tereshchenko S, Verma S, Held C, DeMets DL, Docherty KF, Jhund PS, Bengtsson O, Sjöstrand M, Langkilde AM, Trial Committees DAPA-HF, Investigators, (2019). Dapagliflozin in patients with heart failure and reduced ejection fraction. N Engl J Med.

[4] Packer M, Anker SD, Butler J, Filippatos G, Pocock SJ, Carson P, Januzzi J, Verma S, Tsutsui H, Brueckmann M, Jamal W, Kimura K, Schnee J, Zeller C, Cotton D, Bocchi E, Böhm M, Choi DJ, Chopra V, Chuquiure E, Giannetti N, Janssens S, Zhang J, Gonzalez Juanatey JR, Kaul S, Brunner-La Rocca H-P, Merkely B, Nicholls SJ, Perrone S, Pina I, Ponikowski P, Sattar N, Senni M, Seronde MF, Spinar J, Squire I, Taddei S, Wanner C, Zannad F, EMPEROR-Reduced Trial Investigators, (2020). Cardiovascular and renal outcomes with empagliflozin in heart failure. N Engl J Med.

[5] Anker SD, Butler J, Filippatos G, Ferreira JP, Bocchi E, Böhm M, Brunner-La Rocca H-P, Choi D-J, Chopra V, Chuquiure-Valenzuela E, Giannetti N, Gomez-Mesa JE, Janssens S, Januzzi JL, Gonzalez-Juanatey JR, Merkely B, Nicholls SJ, Perrone SV, Piña IL, Ponikowski P, Senni M, Sim D, Spinar J, Squire I, Taddei S, Tsutsui H, Verma S, Vinereanu D, Zhang J, Carson P, Lam CSP, Marx N, Zeller C, Sattar N, Jamal W, Schnaidt S, Schnee JM, Brueckmann M, Pocock SJ, Zannad F, Packer M, EMPEROR-Preserved Trial Investigators, (2021). Empagliflozin in heart failure with a preserved ejection fraction. N Engl J Med.

[6] Verma S, McMurray JJV (2018). SGLT2 inhibitors and mechanisms of cardiovascular benefit: a state-of-the-art review. Diabetologia.

[7] Karwi QG, Biswas D, Pulinilkunnil T, Lopaschunk GD (2020). Myocardial ketones metabolism in heart failure. J Cardiac Fail.

[8] Fonseca-Correa JI, Correa-Rotter R (2021). Sodium-glucose cotransporter 2 inhibitors mechanisms of action: a review. Front Med.

[9] Sano M (2018). A new class of drugs for heart failure: SGLT2 inhibitors reduce sympathetic overactivity. J Cardiol.

[10] Scheen AJ (2019). Effect of SGLT2 inhibitors on the sympathetic nervous system. Curr Cardiol Rep.

[11] Herat LY, Magno AL, Rudnicka C, Hricova J, Carnagarin R, Ward NC, Arcambal A, Kiuchi MG, Head GA, Schlaich MP, Matthews VB (2020). SGLT2 inhibitor-induced sympathoinhibition: a novel mechanism for cardiorenal protection. JACC Basic Transl Sci.

[12] Gueguen C, Burke SL, Barzel B, Eikelis N, Watson AMD, Jha JC, Jackson KL, Sata Y, Lim K, Lambert GW, Jandeleit-Dahm KAM, Cooper ME, Thomas MC, Head GA (2020). Empagliflozin modulates renal sympathetic and heart rate baroreflexes in a rabbit model of diabetes. Diabetologia.

[13] Ni L, Yuan C, Chen G, Zhang C, Wu X (2020). SGLT2i: beyond the glucose-lowering effect. Cardiovasc Diabetol.

[14] Vrhovac I, Eror DB, Klessen D, Burger C, Breljak D, Kraus O, Radović N, Jadrijević S, Aleksic I, Walles T, Sauvant C, Sabolić I, Koepsell H (2015). Localications of Na^+^-D-glucose cotransporters SGLT1 and SGLT2 in human kidney and of SGLT1 in human small intestine, liver, lung, and heart. Pflugers Arch Eur J Physiol.

[15] Kopp UC (2015). Role of renal sensory nerves in physiological and pathophysiological conditions. Am J Physiol Regul Integr CompPhysiol.

[16] Kopp UC (2018) Neural control of renal function. 2nd edn. Morgan & Claypool Life Sciences 21850765

[17] Yamamoto S, Yamamoto M, Nakamura J, Mii A, Yamamoto S, Takahashi M, Kaneko K, Uchino E, Sato Y, Fukuma S, Imamura H, Matsuda M, Yanagita M (2020). Spatiotemporal ATP dynamics during AKI predict renal prognosis. J Am Soc Nephrol.

[18] Mather A, Pollock C (2011). Glucose handling by the kidney. Kidney Int.

[19] Kawada T, Yamamoto H, Yokoi A, Nishiura A, Kakuuchi M, Yokota S, Matsushita H, Alexander J, Saku K (2023). Acute effects of empagliflozin on open-loop baroreflex function and urine glucose excretion in Goto-Kakizaki diabetic rats. J Physiol Sci.

[20] Kawada T, Sugimachi M (2016). Open-loop static and dynamic characteristics of the arterial baroreflex system in rabbits and rats. J Physiol Sci.

[21] Kawada T, Nishikawa T, Hayama Y, Li M, Zheng C, Uemura K, Saku K, Miyamoto T, Sugimahic M (2021). Quantitative assessment of the central versus peripheral effect of intravenous clonidine using baroreflex equilibrium diagrams. J Physiol Sci.

[22] Damman K, Testani JM (2015). The kidney in heart failure: an update. Eur Heart J.

[23] Mento PF, Maita ME, Wilkes BM (1996). Renal hemodynamics in rats with myocardial infarction: selective antagonism of angiotensin receptor subtypes. Am J Physiol.

[24] Kawada T, Hayama Y, Nishikawa T, Suehara S, Sawada S, Tanaka T, Uenohara M, Sugimachi M (2020). Open-loop analysis on sympathetically mediated arterial pressure and urine output responses in rats: effect of renal denervation. J Physiol Sci.

[25] Li M, Zheng C, Sato T, Kawada T, Sugimachi M, Sunagawa K (2004). Vagal nerve stimulation markedly improves long-term survival after chronic heart failure in rats. Circulation.

[26] Fudim M, Ponikowski PP, Burkhoff D, Dunlap ME, Sobotka PA, Molinger J, Patel MR, Felker GM, Hernandez AF, Litwin SE, Borlaug BA, Bapna A, Sievert H, Reddy VY, Engelman ZJ, Shah SJ (2021). Splanchnic nerve modulation in heart failure: mechanistic overview, initial clinical experience, and safety considerations. Eur J Heart Fail.

[27] Shoukas AA, Callahan CA, Lash JM, Haase EB (1991). New technique to completely isolate carotid sinus baroreceptor regions in rats. Am J Physiol.

[28] Sato T, Kawada T, Miyano H, Shishido T, Inagaki M, Yoshimura R, Tatewaki T, Sugimachi M, Alexander J, Sunagawa K (1999). New simple methods for isolating baroreceptor regions of carotid sinus and aortic depressor nerves in rats. Am J Physiol.

[29] Kawada T, Yokoi A, Nishiura A, Kakuuchi M, Li M, Uemura K, Suehara S, Sawada S, Saku K (2023). Impact of neurally mediated antidiuretic effect relative to pressure diuresis during acute changes in sympathetic nerve activity. Am J Physiol Regul Integr Comp Physiol.

[30] Kent BB, Drane JW, Blumenstein B, Manning JW (1972). A mathematical model to assess changes in baroreceptor reflex. Cardiology.

[31] Mohrman D, Heller L (2010). Cardiovascular Physiology.

[32] Sato T, Kawada T, Inagaki M, Shishido T, Takaki H, Sugimachi M, Sunagawa K (1999). New analytic framework for understanding sympathetic baroreflex control of arterial pressure. Am J Physiol.

[33] Kawada T, Nishikawa T, Suehara S, Sawada S, Tanaka T, Uenohara M, Yamamoto H, Sugimachi M (2021). Open-loop analysis on sympathetically mediated arterial pressure and urine output responses in spontaneously hypertensive rats: effect of renal denervation. J Physiol Sci.

[34] Kawada T, Li M, Suehara S, Sawada S, Zheng C, Uemura K, Sugimachi M, Saku K (2022). Angiotensin II inhibition increases diuresis during acute sympathetic activation in intact and denervated kidneys in rats with chronic myocardial infarction. Heart Vessels.

[35] Glantz SA (2012). Primer of Biostatistics.

[36] Katholi RE, McCann WP, Woods WT (1985). Intrarenal adenosine produces hypertension via renal nerves in the one-kidney, one clip rat. Hypertension.

[37] Hasan A, Hasan R (2021). Empagliflozin relaxes resistance mesenteric arteries by stimulating multiple smooth muscle cell voltage-gated K^+^ (K_V_) channels. Int J Mol Sci.

[38] Yokoi A, Kawada T, Yokota S, Kakuuchi M, Matsushita H, Nishiura A, Li M, Uemura K, Alexander J, Tanaka R, Saku K (2023). Impact of vericiguat on baroreflex-mediated sympathetic circulatory regulation: an open-loop analysis. PLoS ONE.

[39] Hallow KM, Helmlinger G, Greasley PJ, McMurray JJV, Boulton DW (2018). Why do SGLT2 inhibitors reduce heart failure hospitalization? A differential volume regulation hypothesis. Diabetes Obes Metab.

[40] Thomas MC, Cherney DZI (2018). The action of SGLT2 inhibitors on metabolism, renal function and blood pressure. Diapetologia.

[41] Inaba Y, Hashiuchi E, Watanabe H, Kimura K, Sato M, Kobayashi M, Matsumoto M, Kitamura T, Kasuga M, Inoue H (2019). Hepatic gluconeogenic response to single and long-term SGLT2 inhibition in lean/obese male hepatic G6pc-reporter mice. Endocrinology.

[42] Zeuthen T, Zeuthen E, MacAulay N (2007). Water transport by GLUT2 expressed in Xenopus laevis oocytes. J Physiol.

[43] Choi J, Matoba N, Setoyama D, Watanabe D, Ohnishi Y, Yasui R, Kitai Y, Oomachi A, Kotobuki Y, Nishiya Y, Pieper MP, Imamura H, Yanagita M, Yamamoto M (2023). The SGLT2 inhibitor empagliflozin improves cardiac energy status via mitochondrial ATP production in diabetic mice. Commun Biol.

[44] Kawada T, Li M, Kamiya A, Shimizu S, Uemura K, Yamamoto H, Sugimachi M (2010). Open-loop dynamic and static characteristics of the carotid sinus baroreflex in rats with chronic heart failure after myocardial infarction. J Physiol Sci.

[45] Uriel N, Sayer G, Annamalai S, Kapur NK, Burkhoff D (2018). Mechanical unloading in heart failure.

[46] Molina-Linde JM, Cordero-Pereda D, Baños-Álvarez E, Rosario-Lozano MP, Blasco-Amaro JA (2023). Efficacy and safety of baroreflex activation therapy for heart failure with reduced ejection fraction: systematic review. ESC Heart Fail.

[47] Jackson EK, Robertson D (2004). Autonomic control of the kidney. Primer on the autonomic nervous system.

[48] Uemura K, Kawada T, Kamiya A, Aiba T, Hidaka I, Sunagawa K, Sugimachi M (2005). Prediction of circulatory equilibrium in response to changes in stressed blood volume. Am J Physiol Heart Circ Physiol.

